# The Physical Activity Questionnaire for the Elderly (PAQE): A Polish Adaptation

**DOI:** 10.3390/ijerph16244947

**Published:** 2019-12-06

**Authors:** Magdalena Król-Zielińska, Monika Ciekot-Sołtysiak, Wiesław Osiński, Adam Kantanista, Jacek Zieliński, Robert Szeklicki

**Affiliations:** 1 Department of Physical Education and Lifelong Sports, Poznan University of Physical Education, Królowej Jadwigi 27/39, 61-871 Poznań, Poland; adam.kantanista@gmail.com; 2 Department of Athletics Strength and Conditioning, Poznan University of Physical Education, Królowej Jadwigi 27/39, 61-871 Poznań, Poland; ciekot@awf.poznan.pl (M.C.-S.); jacekzielinski@wp.pl (J.Z.); 3 Department of Physical Activity Sciences and Health Promotion, Poznan University of Physical Education, Królowej Jadwigi 27/39, 61-871 Poznań, Polandszeklicki@awf.poznan.pl (R.S.)

**Keywords:** physical activity measurement, older adults, validity, reliability

## Abstract

The aim of the study is to assess the reliability and validity of the Polish adaptation of the Physical Activity Questionnaire for the Elderly (PAQE-PL). One hundred and four older adults (75 women and 29 men) aged 65 to 89 (mean 72.2 ± 5.7 years) participated in the study. The test–retest procedure was used to evaluate the reliability of the PAQE-PL. Validity was assessed by comparing the results of the PAQE-PL with the measurements from an accelerometer (ActiGraph wGT3X+) and two questionnaires: the Polish version of the Community Healthy Activities Model Program for Seniors (CHAMPS-PL) and the Polish version of the Yale Physical Activity Survey (YPAS-PL). All test–retest interclass correlation coefficients (ICCs) were significant (ranged from 0.64 to 0.92). The long-term stability showed significant ICCs (ranged from 0.38 to 0.87) for all participants. In regard to validity, the obtained correlation coefficients were relatively low but statistically significant for all participants between the PAQE-PL scores and energy expenditure (r ranging from 0.25 to 0.26) measured by the accelerometer. The PAQE-PL correlated with almost all CHAMPS-PL indices, YPAS-PL energy expenditure, and total physical activity time. The results suggest that the adaptation of the PAQE-PL is an acceptable tool to estimate the physical activity level among older adults in the Polish population. We recommend the cautious and well-thought-out use of the PAQE-PL with a population of older women.

## 1. Introduction

The health benefits resulting from appropriate physical activity among older adults are indisputable [1]. However, the rates of physical inactivity and sedentariness among older adults are high and related to health consequences, such as functional decline and disabilities, shortened life expectancy, cardiovascular disease, diabetes, and cancer [2]. Due to the aging of societies, research is focusing on how to improve the quality of life among older adults. In this regard, physical activity seems to be one of the best solutions.

To identify relationships between physical activity and health outcomes, a valid and reliable measurement of physical activity is necessary. Methodologically strong studies have shown even larger associations between physical activity and health [3]. An accurate tool also helps to identify the mechanisms of such relationships [4].

Questionnaires are the most commonly used research tools for the measurement of physical activity levels. So-called subjective (self-report) methods have some limitations, but they are not expensive, easily accepted by respondents, not complicated to use, nonreactive, and allow researchers to obtain a large amount of data in a short time [5]. It is likely there are differences between the measurement of physical activity among older adults in comparison to the measurement among other age groups (e.g., specific types of physical activity, stronger seasonal differentiation).

Among different questionnaires, the Physical Activity Questionnaire for the Elderly (PAQE) provides quantitative data related to habitual physical activity (including household, sports, and leisure activities) during the last year [6]. The PAQE is also known as the “modified-Baecke questionnaire” because Voorrips [6] adapted the physical activity questionnaire of Baecke [7] for older adults.

In comparison to other questionnaires, the PAQE has relatively high scores of validity and reliability and is recommended as a useful and credible tool for use in epidemiologic and intervention studies [8], but the number of validation studies is low [9]. Voorrips [6] used the repeated 24-h activity recall and pedometer to estimate the PAQE validity and obtained Spearman’s correlation coefficients of 0.78 and 0.73, respectively. Additionally, the test–retest reliability after 20 days was 0.89. The PAQE was used as a concurrent tool in the validation of the “Assessment of Physical Activity in Frail Older People” (APAFOP) questionnaire among older people with and without cognitive impairment [10]. During this study, the PAQE obtained moderate and good statistically significant correlation coefficients of 0.46 in the total group, 0.31 among cognitively impaired individuals, and 0.64 among cognitively intact individuals based on objective measurement (Physilog system containing two accelerometers and one gyroscope). Hertogh et al. [11] used doubly labeled water in a validation study of the PAQE. The identified relationship was 0.54, and the results showed the correct individual classification of low and high levels of physical activity but poor validity in the case of moderate activity.

In comparison to youth and adults, the number of questionnaires to assess the physical activity of older adults is relatively low [12]. Additionally, in Poland, there were no questionnaires adapted for the population above 69 years, which impeded research on physical activity among older adults and made it difficult to make reliable comparisons with other populations. Although the International Physical Activity Questionnaire was adapted, it was suitable for people aged 15–69 [13]. Therefore, there was a need for research that, according to the methodological requirements of scientific research, created valid and reliable questionnaires for older adults adapted to the Polish context.

In view of the above arguments and to fill the gap in the absence of relevant studies, we implemented a project where the aim was to adapt three questionnaires of physical activity for older adults to the Polish conditions: the Community Healthy Activities Model Program for Seniors (CHAMPS) [14], the Yale Physical Activity Survey (YPAS) [15], and the Physical Activity Questionnaire for the Elderly (PAQE) [6]. Results of research on the Polish adaptation of CHAMPS-PL [16] and the YPAS-PL [17] have been published. This article presents the process of the Polish adaptation of the PAQE.

The aim of the study is to assess the reliability (test–retest within 1 week, and after 3, 6, and 9 months) and validity (by comparing with two questionnaires and an accelerometer) of the Polish adaptation of the Physical Activity Questionnaire for the Elderly (PAQE-PL). The assumption was made that the PAQE-PL is an accurate and credible tool for physical activity measurement in older Polish adults, and values of validity and reliability indicators would be similar to those obtained in previous studies.

## 2. Materials and Methods

### 2.1. Participants and Procedure

In this study of the PAQE-PL, 104 older adults (75 women and 29 men) aged 65 to 89 were included. All participants were volunteers who were invited to participate in research by announcements in the local press, the website of the Poznan University of Physical Education, information disseminated by the Centre for Senior Citizens Initiatives, and leaflets distributed in senior organizations and pharmacies. Only people aged 65+ and able to move independently could participate in the study. Detailed information about the research was presented to the participants, and written consent was obtained. The objectives and study methodology were approved by the ethics committee at the Poznan University of Medical Sciences (971/12).

### 2.2. PAQE

The PAQE designed by Voorrips et al. [6] is based on the activity questionnaire created by Baecke et al. [7]. Instead of occupational activity, which was used in the Baecke questionnaire, household activities were incorporated into the PAQE. In the questionnaire, respondents are asked about their habitual physical activity during the last year, concerning household, sport and other leisure time activities. There are four to five possible answers for household activity questions, ranging from very active (4 points) to inactive (0 points). The household score is the sum of all ten items divided by 10. With regard to sports and other leisure activities, information about the activity type, hours per week, and period of the year in which the activity is normally performed is obtained. The intensity codes (based on energetic costs of activities) are used to characterize the type of leisure and sport activities. Additionally, codes are provided for hours of the week and periods of the year. The sport and other leisure activity scores (points) are the result of the multiplication of the three codes mentioned above. The total result (points) is the sum of all activity domains. Approximately 30 min is necessary to complete the questionnaire.

### 2.3. Polish PAQE Adaptation Procedure–Translation

Before translation, the purpose and structure of the PAQE were considered. Then, the text was translated into Polish by two independent bilingual persons. Both translated versions were compared, discussed, and a unified draft version was prepared. Next, the questionnaire was retranslated into English by two other bilingual persons. The revision and comparison were repeated, and the final Polish version (PAQE-PL) was prepared (see Supplementary Materials S1).

### 2.4. Evaluation of the Reliability and Validity of the Polish Adaptation of the PAQE-PL

According to the purposes of this study, research on the validity and test–retest reliability was conducted. To assess the reliability aspect of the adapted PAQE-PL version, recurrent measurement was performed (repeated measurement with the use of the same test—test-retest within 1 week—tests time stability). Additionally, since the questionnaire refers to annual physical activity, the longer time stability of the PAQE-PL was also determined after 3, 6, and 9 months.

For validity verification, the results of the PAQE-PL were compared to scores obtained from objective physical activity measurement with the use of the ActiGraph accelerometer, model wGT3X+ (ActiGraph, LLC, Pensacola, FL, USA). The ActiGraph was placed on the anterior superior iliac spine (on the belt around the waist). The measurement lasted for 7 consecutive days (all day, with exception of sleeping time, bathing, showering, or swimming) and recorded data in 10 s time epochs. The ActiLife6 analysis software suite (ActiGraph, LLC, Pensacola, FL, USA) was used for data analysis. After the 7-day measurement of physical activity by the accelerometer, the PAQE-PL was completed. The weekly energy expenditure obtained from the accelerometer was calculated using Freedson’s equation [18]. Wear-time was assessed by ActiLife using the wear-time validation module. The wear-time in minutes was calculated from the algorithm [19]. Participants who did not fulfil the criteria of 600 min accelerometer wearing-time per day [20] were excluded from the analysis. The unit used for accelerometer energy expenditure was kilocalories per week. The units for sedentary, moderate activity, vigorous activity, and moderate-to-vigorous physical activity time were hours per week.

The CHAMPS-PL [14,16] assessed caloric expenditure (kilocalories per week) and frequency (per week) for all activities (any metabolic equivalent (MET) value) and at least moderate-intensity activities (MET value ≥3.0). When completing the CHAMPS-PL physical activity questionnaire, respondents had to recall the type and frequency of physical activities undertaken during one typical week in the past four weeks.

The YPAS-PL [15,17] assessed energy expenditure (kilocalories per week) and the time devoted to physical activity (hours per week) during housework, yardwork, caretaking, exercise, and recreational activities. The recall period applied to a typical week in the month preceding the study.

### 2.5. Statistical Analyses

STATISTICA 13 software (StatSoft, Inc., Tulsa, OK, USA) was used for all analyses, except for the calculation of interclass correlation coefficients (ICCs), which was performed using Predictive Analytics Software (PASW) v.18.0 (IBM Corp., Armonk, NY, USA). The basic statistical characteristics (mean, standard deviation, range) were calculated for quantitative variables, and the percentage distribution was estimated to describe the qualitative variables. Gender differences for continuous (anthropometric) variables were calculated using a *t*-test and the structure indicator was used for nominal (demographic) variables. ICCs were calculated to assess the reliability, and Pearson’s correlation was used to estimate the validity of the PAQE-PL. The interpretation of ICCs was based on the criteria of Koo and Li [21]: below 0.50 is poor, between 0.50 and 0.75 is moderate, between 0.75 and 0.90 is good, and above 0.90 is excellent. After Terwee et al. [22], we assumed acceptable reliability of the physical activity questionnaire if the ICC value was above 0.70. In the interpretation of correlation coefficients values, Cohen’s [23] classification was used. A correlation coefficient of 0.10 indicated a small relationship, and values of 0.30 and 0.50 were considered medium and large correlations, respectively. After Terwee et al. [22], we assumed the physical activity questionnaire was valid if the correlation was above 0.50 for accelerometer and 0.30 for other questionnaires.

## 3. Results

The sample included 104 older adults (75 women and 29 men) aged 65 to 89 (mean age 72.2 ± 5.7 years). The average body height of the women was 1.59 ± 0.06 m, and 1.70 ± 0.06 m for men. The average body weight was 67.8 ± 12.6 kg in women and 80.3 ± 9.6 kg in men. The average body mass index (BMI) was 27.0 ± 3.9 kg/m^2^ for all participants, 26.7 ± 4.0 kg/m^2^ in women and 27.8 ± 3.6 kg/m^2^ in men. There were significant differences between men and women in body weight and height (*p* < 0.001), but there were no gender differences in BMI (*p* = 0.219). The participants’ basic demographic characteristics are presented in Table 1.

Table 2 presents the results of the PAQE-PL test-retest reliability within 1 week and after 3, 6, and 9 months. All the results of 1 week time stability ICCs were significant (*p* < 0.001) and ranged from moderate (0.64) to excellent (0.92) for all the PAQE-PL outcomes. The highest repeatability rate was observed for household activities (ICC = 0.92) among women. In the group of men, all test–retest correlation coefficients were similar and high (ICC = 0.85 and ICC = 0.86). The results of the long-term stability research of the PAQE-PL after 3, 6, and 9 months for all participants were significant and mostly moderate. In the group of women, the ICCs ranged from poor to moderate, and the leisure time activities and overall questionnaire scores were mostly not statistically significant. Only for household activities were the reliability indicators significant, but they decreased in each successive time point of test–retests. Among men, all ICCs were statistically significant (mostly *p* < 0.001) and ranged from moderate (r = 0.71) to excellent (r = 0.95).

Table 3 shows the results of the validity of the PAQE-PL. The accelerometer outcomes (energy expenditure, sedentary time, moderate physical activity time, vigorous physical activity time, and moderate-to-vigorous physical activity time) during the one week of physical activity were compared to the habitual physical activity in the last year assessed by the PAQE-PL. The obtained correlation coefficients (ranging from 0.25 to 0.26) between the PAQE-PL scores and the energy expenditure measured by the accelerometer were statistically significant for all participants. Leisure time activities and overall PAQE-PL score were significantly related to moderate activity time assessed by accelerometer among all individuals (r = 0.27 and r = 0.28, respectively). Moreover, the accelerometer-assessed vigorous activity time of women correlated significantly with all the PAQE-PL scores (ranging from 0.27 to 0.33). Among men, only PAQE-PL household activities correlated significantly with energy expenditure (r = 0.55), moderate activity time (r = 0.45) and moderate-to-vigorous physical activity (r = 0.45) measured by the accelerometer.

Validity was also assessed by comparing the results of the PAQE-PL with subjective measures of physical activity (CHAMPS-PL and YPAS-PL; see Table 4 and Table 5, respectively). The PAQE-PL scores were significantly related to almost all parameters of physical activity measured by the CHAMPS-PL (ranging from 0.19 to 0.40) in all participants (Table 4). Leisure time activity and the overall score of the PAQE-PL of women were significantly correlated with all CHAMPS-PL indices (ranging from 0.37 to 0.40). Household activities measured by the PAQE-PL did not correlate with any CHAMPS-PL score among women. Most of the PAQE-PL scores of men correlated with physical activity, as measured by the CHAMPS-PL.

Significant correlations were found between the overall PAQE-PL score and YPAS-PL energy expenditure (r = 0.32) and total physical activity time (r = 0.26) in all individuals (Table 5). Similarly, the PAQE-PL leisure time activity score was also correlated with all the YPAS-PL parameters (ranging from 0.23 to 0.30). The household activity score of the PAQE-PL was significantly correlated with energy expenditure (r = 0.42) and total physical activity time (r = 0.43) measured by the YPAS-PL among all participants. Among women, the PAQE-PL results correlated significantly with the YPAS-PL only in the context of household activity. Among men, almost all PAQE-PL scores correlated with YPAS-PL indices.

## 4. Discussion

In this study, the validity and reliability of the Polish adaptation of the PAQE-PL are presented. To date, there have been no other studies related to a Polish version of the PAQE.

Relatively high one-week test–retest indicators were obtained in this study, especially among men. Similar results were presented by Voorrips et al. [6] in repeated measurements after 20 days. Martin et al. [24] indicated that men with advancing age increased their sedentary behavior and reduced their moderate-to-vigorous physical activity time. They also suggested that women continue engaging in common daily activities. Montoye et al. [25] showed that it is difficult for older people to recall physical activity accurately. The reason for the higher test–retest indicators among men could be simply that they have less to recall.

A similar explanation of the obtained results, as discussed above, can be used for the interpretation of the long-term (after 3, 6, and 9 months) reliability. The ICCs were lower in comparison to the one-week test–retest indicators but were still satisfactory. Again, much higher ICCs were obtained among men. The PAQE-PL estimates one-year physical activity, but especially among older adults, seasonal differences exist in regard to the level of physical activity [26]. Measurements were repeated every three months in different seasons. It seems to be natural that the evaluation of the entire year’s physical activity can be influenced by its present level.

The comparison of the obtained validation results with those of other studies is difficult because of the limited available research [9]. In our study, the validity indicators were significant but lower than those from the Voorrips’ [6] study, where the repeated 24-h activity recall and pedometer were included instead of the accelerometer. Similarly, Mazo et al. [27] used a pedometer in the validation study of PAQE and also showed relatively low correlation coefficient value (r = 0.27) among 30 elderly women in Brazil. The accelerometer was used as a validation tool by Hauer et al. [10], who also obtained a higher correlation coefficient in the group of cognitively intact participants. Additionally, Hertogh et al. [11] showed higher indicators of validity in comparison to the doubly labeled water, which was used as a criterion. The potential explanation of the relatively low correlation coefficient values that were obtained could be the fact that important components of the PAQE-PL are household activities. During some household activities, acceleration is low, which decreases the physical activity estimated by the accelerometer [28]. Moreover, in the study of Machado et al. [29], it was suggested that Freedson’s energy expenditure algorithm, that was used in our study, is not sensitive to low-intensity activity measurements. Considering that older adults are characterized by mainly low-intensity activities, this could be another potential explanation of the low association between the results received by PAQE-PL and the accelerometer scores.

The comparison of the obtained results between the PAQE-PL and the CHAMPS-PL, as well as between the PAQE-PL and the YPAS-PL, showed mostly significant and moderately high correlation coefficients. The proper interpretation must include the specificity of the CHAMPS-PL and the YPAS-PL questionnaires. The CHAMPS-PL is intended to evaluate the effect of a physical activity promotion intervention. Physical activity promotion includes leisure-time and sports activities. This difference could be the reason for the very low and even nonsignificant (among women) correlations among household activities. A similar situation exists when the PAQE-PL outcomes are compared to values obtained from the accelerometer. In contrast, the YPAS-PL is intended to measure the age-specific physical activities of older adults, of which household activities are very important. Consequently, among women, a statistically significant correlation was found only for household activities.

Also, Troiano et al. [30] reported that low correlations are observed between self-report and accelerometer assessed PA because these methods are “not equivalent”. Kelly et al. [31] critically assessed existing validation studies, but there is no perfect solution since physical activity has many aspects and cannot be measured using a single method.

In the final interpretation of this study, some limitations should be considered. Firstly, only volunteers who presented a good health state and who were usually more physically and socially active participated in the study. Secondly, the participants were mostly women, which is a weakness in terms of drawing a general conclusion. Thirdly, the systematic completion of the questionnaire could increase the participants’ awareness and perception of their own physical activity, which could finally influence the reliability of measurement.

It is worth emphasizing the advantages of this study. A modern accelerometer was used as a tool in the estimation of construct validity. Moreover, except for the simple test–retest research, long-term reliability was estimated, which has not yet been recognized in research.

## 5. Conclusions

A general overview of the obtained results suggests that the PAQE-PL is an acceptable tool to estimate physical activity levels among older adults in the Polish population. We recommend the cautious and well-thought-out use of the PAQE-PL with a population of older women.

## Figures and Tables

**Table 1:** Demographic characteristics of participants and gender differences.

Demographic Variables	Total *n* = 104		Women *n* = 75		Men *n* = 29		Gender Differences
	*n*	%	*n*	%	*n*	%	*p*
**Marital status**							
Married	55	52.9	31	41.3	24	82.8	<0.001
Widow/widower	32	30.8	29	38.7	3	10.3	0.005
Divorced	10	9.6	8	10.7	2	6.9	0.556
Unmarried	7	6.7	7	9.3	0	0	0.089
**Education**							
Higher	47	45.6	31	41.9	16	55.2	0.222
Secondary	35	34.0	30	40.5	5	17.2	0.025
Vocational	13	12.6	6	8.1	7	24.1	0.027
Primary	8	7.8	7	9.5	1	3.5	0.307
**Place of residence**							
Fewer than 20,000 inhabitants	22	21.1	16	21.3	6	20.7	0.946
20,000–500,000 inhabitants	14	13.5	10	13.3	4	13.8	0.947
More than 500,000 inhabitants	68	65.4	49	65.4	19	65.5	0.992

**Table 2:** ICCs of the time stability (test–retest within 1 week) and the long-term stability (test–retest after 3, 6, and 9 months) of the PAQE-PL.

PAQE-PL	Test-Retest			
	After 1 Week	After 3 Months	After 6 Months	After 9 Months
**All participants**				
Questionnaire score	0.73 ***	0.66 ***	0.63 ***	0.64 ***
Household activities	0.91 ***	0.87 ***	0.78 ***	0.62 ***
Leisure time activities	0.73 ***	0.38 *	0.63 ***	0.65 ***
**Women**				
Questionnaire score	0.64 ***	0.46 **	0.35	0.37
Household activities	0.92 ***	0.74 ***	0.64 ***	0.45 *
Leisure time activities	0.64 ***	0.03	0.35	0.39 *
**Men**				
Questionnaire score	0.86 ***	0.92 ***	0.95 ***	0.91 ***
Household activities	0.85 ***	0.92 ***	0.88 ***	0.76 **
Leisure time activities	0.86 ***	0.71 **	0.95 ***	0.90 ***

* *p* < 0.05, ** *p* < 0.01, *** *p* < 0.001.

**Table 3:** Correlation coefficients between the PAQE-PL scores and accelerometer measurements.

PAQE-PL	Accelerometer				
	Energy Expenditure	Sedentary Time	Moderate Activity Time	Vigorous Activity Time	Moderate-to-Vigorous Physical Activity Time
**All participants**					
Questionnaire score	0.26 *	−0.14	0.28 *	0.17	0.28 *
Household activities	0.25 *	0.01	0.21	0.19	0.21
Leisure time activities	0.25 *	−0.15	0.27 *	0.17	0.27 *
**Women**					
Questionnaire score	0.29 *	−0.15	0.23	0.33 *	0.24
Household activities	0.18	0.10	−0.01	0.27 *	0.01
Leisure time activities	0.29 *	−0.15	0.24	0.32 *	0.24
**Men**					
Questionnaire score	0.21	−0.15	0.35	−0.20	0.35
Household activities	0.55 *	−0.01	0.45 *	0.11	0.45 *
Leisure time activities	0.19	−0.16	0.33	−0.20	0.33

* *p* < 0.05.

**Table 4:** Correlation coefficients between physical activity measured by the PAQE-PL and the CHAMPS-PL.

PAQE-PL	CHAMPS-PL			
	Frequency of All Listed Activities	Caloric Expenditure of All Listed Activities	Frequency of at Least Moderate-Intensity Activities	Caloric Expenditure of at Least Moderate-Intensity Activities
**All participants**				
Questionnaire score	0.38 ***	0.37 ***	0.40 ***	0.37 ***
Household activities	0.24 *	0.19 *	0.20 *	0.19
Leisure time activities	0.37 ***	0.37 ***	0.40 ***	0.37 ***
**Women**				
Questionnaire score	0.39 ***	0.41 ***	0.38 ***	0.36 **
Household activities	0.15	0.19	0.18	0.22
Leisure time activities	0.39 ***	0.40 ***	0.37 ***	0.35 **
**Men**				
Questionnaire score	0.36 *	0.33	0.48 **	0.43 *
Household activities	0.43 *	0.36 *	0.47 **	0.40 *
Leisure time activities	0.33	0.31	0.46 *	0.41 *

* *p* < 0.05, ** *p* < 0.01, *** *p* < 0.001.

**Table 5:** Correlation coefficients between physical activity measured by the PAQE-PL and the YPAS-PL.

PAQE-PL	YPAS-PL	
	Energy Expenditure	Total Physical Activity Time
**All participants**		
Questionnaire score	0.32 ***	0.26 **
Household activities	0.42 ***	0.43 ***
Leisure time activities	0.30 **	0.24 *
**Women**		
Questionnaire score	0.19	0.13
Household activities	0.40 ***	0.39 ***
Leisure time activities	0.17	0.11
**Men**		
Questionnaire score	0.53 **	0.49 **
Household activities	0.38 *	0.35
Leisure time activities	0.51 **	0.47 **

* *p* < 0.05, ** *p* < 0.01, *** *p* < 0.001.

## Supplementary Materials

The following are available online at https://www.mdpi.com/1660-4601/16/24/4947/s1, S1: PAQE-PL.
