# Supplementary material for: The Physical Activity Questionnaire for the Elderly (PAQE): A Polish Adaptation
Source: Int J Environ Res Public Health. 2019 Dec 6;16(24):4947. doi: 10.3390/ijerph16244947 (PMC6950819; doi:10.3390/ijerph16244947)
Supplement: Supplementary file 1 [file ijerph-16-04947-s001.pdf]

PAQE-PL: KWESTIONARIUSZ AKTYWNOŚCI FIZYCZNEJ U OSÓB STARSZYCH - POLSKA ADAPTACJA (ORYGINALNA WERSJA: PAQE)

PRACE DOMOWE

1. Czy wykonuje Pan/i lekkie prace domowe? (ścieranie kurzu, mycie naczyń, naprawa ubrań, itp.)? ☐
   0. Nigdy (mniej niż raz w miesiącu)
   1. Czasami (tylko kiedy partner lub pomoc domowa nie może)
   2. Zazwyczaj (czasami z asystą partnera lub pomocą domową)
   3. Zawsze (samodzielnie lub razem z partnerem)

2. Czy wykonuje Pan/i ciężkie prace domowe? (mycie podłóg i okien, wynoszenie śmieci, itp.)? ☐
   0. Nigdy (mniej niż raz w miesiącu)
   1. Czasami (tylko kiedy partner lub pomoc domowa nie może)
   2. Zazwyczaj (czasami z asystą partnera lub pomocą domową)
   3. Zawsze (samodzielnie lub razem z partnerem)

3. Dla ilu osób prowadzi Pan/i dom? (wliczając siebie; proszę wpisać „0”, jeśli odpowiedział/a Pan/i „nigdy” w pytaniach 1 i 2) ☐

4. Ile pomieszczeń Pan/i sprząta, wliczając kuchnię, sypialnię, garaż, piwnicę, łazienkę, itd.? (proszę wpisać „0”, jeśli odpowiedział/a Pan/i „nigdy” w pytaniach 1 i 2) ☐
   0. Nigdy nie sprzątam
   1. 1-6 pomieszczeń
   2. 7-9 pomieszczeń
   3. 10 i więcej pomieszczeń

5. Na ilu piętrach znajdują się sprzątane przez Pana/Panią pomieszczenia? (proszę wpisać „0”, jeśli odpowiedział/a Pan/i „nigdy” w pytaniu 4) ☐

6. Czy przygotowuje Pan/i samodzielnie ciepłe posiłki lub uczestniczy Pan/i w ich przygotowaniu? ☐
   0. Nigdy
   1. Czasami (raz lub dwa razy w tygodniu)
   2. Zazwyczaj (3-5 razy w tygodniu)
   3. Zawsze (więcej niż 5 razy w tygodniu)

7. Ile kondygnacji pokonuje Pan/i dziennie wchodząc po schodach? (1 kondygnacja to 10 stopni) ☐
   0. Nigdy nie chodzę po schodach
   1. 1-5
   2. 6-10
   3. Więcej niż 10

8. Jeśli porusza się Pan/i po mieście, to z jakiego środka transportu Pan/i korzysta? ☐
   0. Nigdy nie wychodzę
   1. Samochód
   2. Transport publiczny
   3. Rower
   4. Pieszo

9. Jak często wychodzi Pan/i po zakupy?
   0. Nigdy lub rzadziej niż raz w tygodniu
   1. Raz w tygodniu
   2. Dwa do czterech razy w tygodniu ☐
   3. Codziennie

10. Jeśli wychodzi Pan/i po zakupy, to z jakiego środka transportu Pan/i korzysta?
   0. Nigdy nie wychodzę po zakupy
   1. Samochód
   2. Transport publiczny ☐
   3. Rower
   4. Pieszo

Wynik aktywności fizycznej podczas wykonywania prac domowych = (Pyt. 1 + Pyt. 2 + … + Pyt. 10)/10

AKTYWNOŚĆ SPORTOWA
Czy uprawia Pan/Pani sport?
Dyscyplina 1: nazwa____
intensywność (kod)________________________________ (1a)
liczba godzin tygodniowo (kod)_____________________ (1b)
okres czasu w roku (kod)___________________________ (1c)
Dyscyplina 2: nazwa____
intensywność (kod)________________________________ (2a)
liczba godzin tygodniowo (kod)_____________________ (2b)
okres czasu w roku (kod)___________________________ (2c)

Wynik aktywności sportowej: $\sum_{i=1}^{2}(ia * ib * ic)$

AKTYWNOŚĆ REKREACYJNA
Czy podejmuje Pan/Pani inne formy aktywności fizycznej?
Aktywność 1: nazwa____
intensywność (kod)________________________________ (1a)
liczba godzin tygodniowo (kod)_____________________ (1b)
okres czasu w roku (kod)___________________________ (1c)

Aktywności od 2 do 6: proszę uzupełnić analogiczne, jak w przypadku Aktywności 1.

Wynik aktywności rekreacyjnej: $\sum_{i=1}^{6}(ia * ib * ic)$

WYNIK KWESTIONARIUSZA = WYNIK AKTYWNOŚCI FIZYCZNEJ PODCZAS WYKONYWANIA PRAC DOMOWYCH + WYNIK AKTYWNOŚCI SPORTOWEJ + WYNIK AKTYWNOŚCI REKREACYJNEJ

Kody:

Kod intensywności[1]:

0) pozycja leżąca, bez obciążenia	kod 0.028
1) pozycja siedząca, bez obciążenia	kod 0.146
2) pozycja siedząca, ruchy rąk lub ramion	kod 0.297
3) pozycja siedząca, ruchy całego ciała	kod 0.703
4) pozycja stojąca, bez obciążenia	kod 0.174
5) pozycja stojąca, ruchy rąk lub ramion	kod 0.307
6) pozycja stojąca, ruchy całego ciała, chód	kod 0.890
7) chód, ruchy rąk lub ramion	kod 1.368
8) chód, ruchy całego ciała, jazda na rowerze, pływanie	kod 1.890

Liczba godzin w ciągu tygodnia:

1) mniej niż 1 godz. tygodniowo	kod 0.5
2) 1,2 > godz. tygodniowo	kod 1.5
3) 2,3 > godz. tygodniowo	kod 2.5
4) 3,4 > godz. tygodniowo	kod 3.5
5) 4,5 > godz. tygodniowo	kod 4.5
6) 5,6 > godz. tygodniowo	kod 5.5
7) 6,7 > godz. tygodniowo	kod 6.5
8) 7,8 > godz. tygodniowo	kod 7.5
9) więcej niż 8 godz. tygodniowo	kod 8.5

Liczba miesięcy w ciągu roku:

1) mniej niż 1 miesiąc w ciągu roku	kod 0.04
2) 1-3 miesiące	kod 0.17
3) 4-6 miesięcy	kod 0.42
4) 7-9 miesięcy	kod 0.67
5) więcej niż 9 miesięcy w ciągu roku	kod 0.92

---

[1]Niemianowane kody intensywności stworzone w oparciu o wydatek energetyczny.
